# Journey mapping as a novel approach to healthcare: a qualitative mixed methods study in palliative care

**DOI:** 10.1186/s12913-021-06934-y

**Published:** 2021-09-04

**Authors:** Stephanie Ly, Fiona Runacres, Peter Poon

**Affiliations:** 1 Faculty of Medicine Nursing and Health Sciences, Monash University, Clayton, VIC Australia; 2 Supportive & Palliative Care Department, McCulloch House, Monash Medical Centre, 246 Clayton Road, VIC 3168 Clayton, Australia; 3 Calvary Health Care Bethlehem, Parkdale, VIC Australia

**Keywords:** Patient journey mapping, Health services research, Palliative care, Illness trajectory, Proactive healthcare, Medical informatics, Patient-centred care

## Abstract

**Background:**

Journey mapping involves the creation of visual narrative timelines depicting the multidimensional relationship between a consumer and a service. The use of journey maps in medical research is a novel and innovative approach to understanding patient healthcare encounters.

**Objectives:**

To determine possible applications of journey mapping in medical research and the clinical setting. Specialist palliative care services were selected as the model to evaluate this paradigm, as there are numerous evidence gaps and inconsistencies in the delivery of care that may be addressed using this tool.

**Methods:**

A purposive convenience sample of specialist palliative care providers from the Supportive and Palliative Care unit of a major Australian tertiary health service were invited to evaluate journey maps illustrating the final year of life of inpatient palliative care patients. Sixteen maps were purposively selected from a sample of 104 consecutive patients. This study utilised a qualitative mixed-methods approach, incorporating a modified Delphi technique and thematic analysis in an online questionnaire.

**Results:**

Our thematic and Delphi analyses were congruent, with consensus findings consistent with emerging themes. Journey maps provided a holistic patient-centred perspective of care that characterised healthcare interactions within a longitudinal trajectory. Through these journey maps, participants were able to identify barriers to effective palliative care and opportunities to improve care delivery by observing patterns of patient function and healthcare encounters over multiple settings.

**Conclusions:**

This unique qualitative study noted many promising applications of the journey mapping suitable for extrapolation outside of the palliative care setting as a review and audit tool, or a mechanism for providing proactive patient-centred care. This is particularly significant as machine learning and big data is increasingly applied to healthcare.

## Introduction

Patterns of healthcare utilisation are evolving in response to the ageing population and increasing burden of chronic disease. There is an urgent need to ensure timely proactive medical care, effective and efficient resource deployment, while averting unnecessary, often distressing, emergency department (ED) presentations, admissions and conveyor belt medicine. A key area of medicine able to address these issues is palliative care.

Central to optimal delivery of palliative care is timely initiation [1–3]. However, differing patient, illness trajectory and clinical factors have resulted in inconsistencies in the degree of care provided [4, 5]. This has subsequently translated into significant variability in palliative care research and limitations in applying international evidence to clinical practice [6]. The utilisation of journey mapping has the potential to address these inconsistencies and to our knowledge, this research is the first of its kind.

Journey mapping is a relatively new approach in medical research that has been adapted from customer service and marketing research [7]. It is gaining increasing recognition for its ability to organise complex multifaceted data from numerous sources and explore interactions across care settings and over time. Medical journey mapping involves creating narrative timelines, by incorporating markers of the patient experience with healthcare service encounters. Integrating diverse components of the patient healthcare journey provides a holistic perspective of the relationships between the different elements that may guide directions for change and service improvement. As medical journey mapping is still in its infancy, there is an absence of literature exploring implementation. Of the existing literature, journey mapping techniques are described mainly in process papers, outlining their potential utility in observing healthcare delivery and patient outcomes [8–12]. However journey mapping paradigms have broader significance across healthcare, especially in an environment for which machine learning, big data and artificial intelligence is maturing.

We aimed to determine whether journey mapping could contribute to the improvement of patient-centred medical research in a palliative care setting and provide new insight into possible “pivot-points” or moments of care that could be altered to improve care delivery. Specifically, we sought to determine whether journey maps were able to assist in capturing a holistic, longitudinal and more integrative patient history whilst outlining healthcare provision and identifying gaps in care.

## Methods

### Study design

**Fig. 1:**
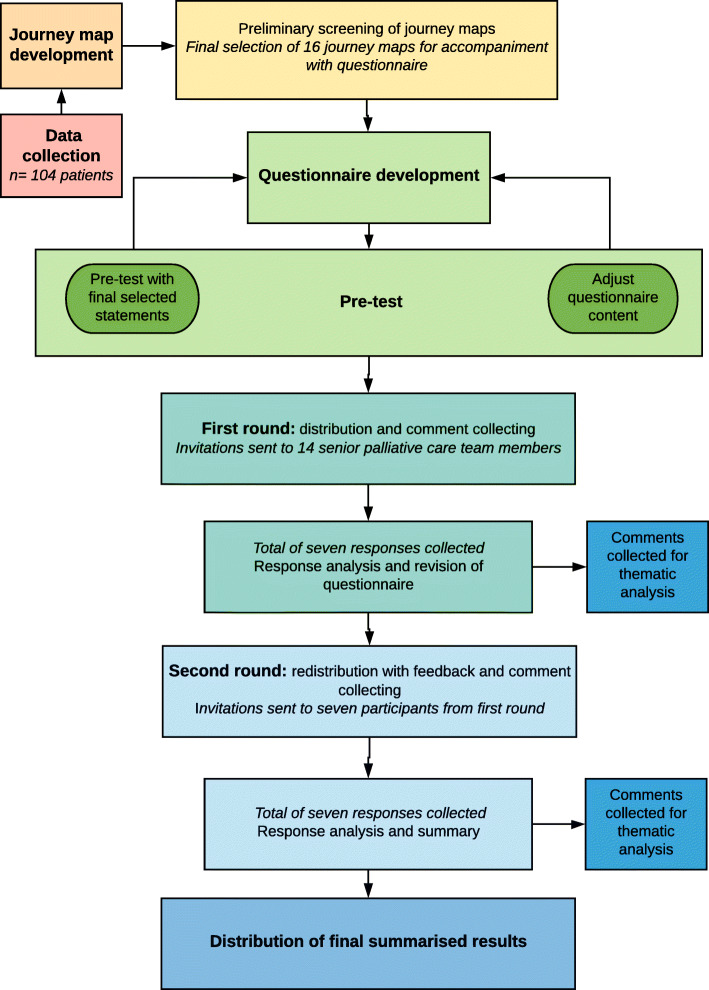
Flow chart detailing data collection, journey map development and analysis. This figure illustrates the phases and processes of this study. Data was collected from a retrospective cohort of 104 palliative care patients and journey maps were subsequently developed. Preliminary screening of the journey maps was performed to obtain a purposive sample that best highlighted the breadth of information and healthcare encounters captured within the journey maps. A total of 16 maps were selected for further analysis. Following questionnaire development and pre-test, questionnaires were distributed, and responses collected and analysed over two rounds to obtain consensus. Free-text comments from both rounds were collected for thematic analysis

We performed a qualitative mixed-methods analysis of a journey mapping tool. The tool was purpose-developed and sample journey maps were derived from the scanned medical records of palliative care patients. A panel of specialist palliative care providers were then involved in an online questionnaire combining a modified Delphi approach with inductive thematic analysis. Figure 1 depicts a flow diagram of the methodology.

All methods were carried out and reported in accordance with Standards for Reporting Qualitative Research (SRQR) guidelines and Consolidated criteria for Reporting Qualitative research (COREQ) criteria for reporting qualitative studies.

### Ethics

Ethics approval for this study was obtained from Monash Health Human Research Ethics Committee Monash Health Ref: RES-29-0000-071Q) and Monash University Human Research Ethics Committee (Project ID: 18,853).

### Data

Data was collected from a retrospective cohort of 104 consecutive palliative care patients from a major tertiary hospital network in Melbourne, Australia. Inclusion criteria were patients greater than 18 years of age who had died in hospital between the 1st of August 2018 and 31st of October 2018, had at least one inpatient palliative care admission in their last year of life and scanned medical records data spanning at least three months’ duration. This sample size was considered sufficient to incorporate a varied and representative sample of palliative care patients encountered in the tertiary hospital.

Following data collection, a Python Software-based code was designed to extract de-identified data and create journey map visuals. All 104 journey maps were independently screened by two investigators (PP and FR) and a purposive sample of 16 maps was selected for analysis based on seven criteria for informative value. The criteria that the 104 maps were assessed on included their ability to provide insight into the initiation, triggers, delivery and barriers of palliative care, SPICT scores, pivot points and disease trajectories.

### Modified Delphi approach and thematic analysis

A qualitative mixed-methods approach involving thematic analysis and a modified Delphi technique was utilised as an explorative analysis of expert opinion. The consensus agreement was used to reinforce and confirm the patterns of significance identified through thematic analysis. In combining these two approaches, there was greater flexibility in responses and additional structure to support analysis.

The modified Delphi approach used in this study was adapted from the enhanced Delphi method described by Yang et al. [13] and consisted of a questionnaire pre-test and two rounds of questionnaire distribution. A total of 14 email invitations were sent to a purposive sample of seven senior palliative care physicians and seven palliative care nurse consultants across two palliative care inpatient units within a major tertiary hospital network in Melbourne, Australia. The emails contained an explanatory statement, a questionnaire link and the file containing the 16 de-identified journey maps.

The questionnaires consisted of 16 statements per journey map, covering eight palliative care domains: palliative care triggers, initiation, delivery, outcomes, barriers, pivot-points, needs assessment (using the Supportive and Palliative Care Indicators Tool, SPICT) and the utility of advanced care plans. An additional nine statements assessed the utility of the journey map approach (see Table 2). All statements were ranked using Likert scales. A four-point Likert scale including the options: *insufficient information, disagree, neither agree nor disagree and agree* was used to assess individual journey maps. A five-point Likert scale including options: *strongly disagree, somewhat disagree, neither agree nor disagree, somewhat agree and strongly agree* was used to assess the journey mapping approach. For analysis of consensus, the results were categorised to reflect overall agreement by using a three-point scale consisting of *disagree, neutral and agree*. Consensus was defined as agreement of greater than 70 % of respondents in any one of these three categories. Following the first round, all consensus statements were determined and participants were sent a second questionnaire containing anonymous feedback from the first round and statements which did not reach consensus for re-evaluation using the condensed three-point Likert scale.

Following each palliative care domain, free-text fields were included to collect comments and provide data for inductive thematic analysis. Analysis of the free-text comments from both rounds was guided by Braun and Clarke’s phases of thematic analysis [14]. The codes and themes were derived from the data using NVivo 12 Plus software to generate nodes, initial codes and preliminary subthemes. Candidate themes were reviewed by two additional investigators (PP and FR) to ensure consistency and the final themes were defined. Providing participants with the opportunity to review de-identified feedback through the Delphi questionnaire enabled discussion, reflection and clarification of comments, thus achieving thematic saturation with a smaller group of participants.

Additional steps were taken to increase trustworthiness of the qualitative data per Lincoln and Guba’s criteria for credibility, transferability, dependability and confirmability across all phases of analysis [15–17]. Triangulation of the methods, researchers and analysts aimed to increase consistency and accuracy, whilst reducing interpretation errors and the effects of bias. Thorough audit trails and reflexive journaling were maintained. The use of the online questionnaire with free-text fields for thematic data collection limited the role of the researcher and the potential for associated bias.

## Results

While this study also produced findings relevant to current issues of palliative care delivery, we will for the purpose of this paper, present results specific to the clinical utility of journey maps.

### The journey maps

**Fig. 2:**
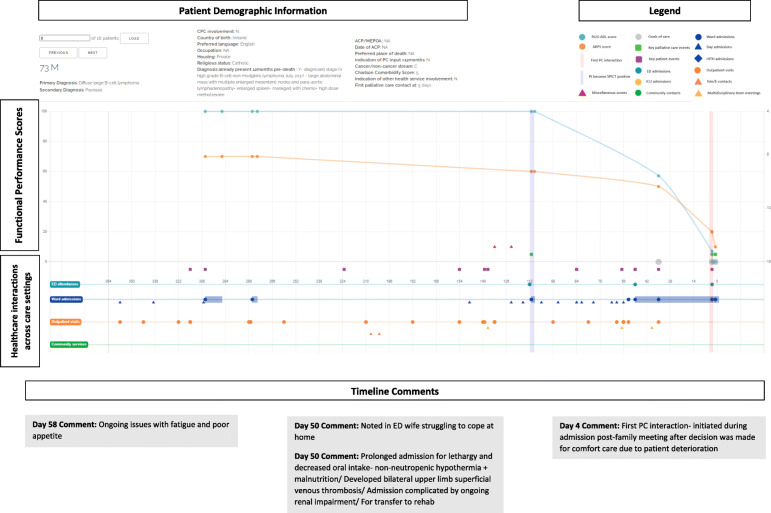
Screen capture of Journey Map 6. A screen capture of one of the 16 interactive journey maps that was analysed by the Delphi panel. The lower segment of the map depicts healthcare interactions that occurred in hospital and in the community. Delphi participants are able to hover over specific health service touch points to obtain more information about the specific interaction that occurred. The upper segment represents functional performance scores using two different tools- the Australian Karnofsky Performance Scale (AKPS) and the Resource Utilisation Groups-Activities of Daily Living (RUG-ADL). The orange vertical line indicates when palliative care needs first presented using the SPICT screening tool. The vertical purple line indicates when specialist palliative care was initiated. Delphi participants were able to analyse the maps and respond to statements on the palliative care provided

Figure 2 depicts one of the 16-sample journey maps analysed by participants and illustrates the key elements of a map. While journey maps are interactive visualisations with options for providing additional information summarising patient healthcare encounters, we are unable to fully convey the dynamic functions of the mapping tool in this paper. The journey map in Fig. 2 illustrates the final year of life of a 73 year old male patient with diffuse large B-cell lymphoma.

The map reveals that at day 112 prior to death, palliative care needs were noted using the SPICT screening tool. It is also at this point that the patient’s functional performance scores began to decline, with patient notes from day admissions and clinic visits also documenting poor tolerance of chemotherapy side effects, fatigue, anorexia and weight loss. In response to this pattern of decline, participants noted that there was an opportunistic role for community palliative care support that was missed and could have potentially negated the need for the final ED admission.

*“Onc (sic)(oncology) outpatient notes describing symptoms, deterioration, carer distress… Community pall care (sic)(palliative care) could have been helpful”* – Participant 2, Journey Map (JM) 6.

Another major pivot-point occurred during the patient’s admission to ED on day 50 when notes indicated that the patient’s wife was struggling to cope with care at home. Given the nature of the prolonged admission with multiple complications that followed, Delphi participants questioned the suitability of the transfer to the rehabilitation ward.

*“Symptoms and functional decline appear to be related to lymphoma and not an acute illness. More appropriate for pall care (sic) than rehab (sic)(rehabilitation).”* – Participant 6, JM6.

Additionally, the decision to initiate palliative care only four days prior to death was delayed and there was a role for earlier palliative care involvement.

*“Clearly PC (sic)(palliative care) involvement inadequate and was a later referral for terminal care only”* – Participant 1, JM6.

*“Pt (sic)(patient) would have benefitted from earlier palliative care referral”* – Participant 7, JM6.

Through the maps, participants were able to observe patterns of deterioration with a broader view of continuity of care and determine pivot-points, where the involvement of specialist palliative care had the potential to improve the patient experience.

### Modified Delphi

The two Delphi rounds were conducted over 31 days with the first round taking 13 days and the second round spanning 18 days. A total of seven responses were collected from the first round of the online Delphi questionnaire. Six members of the medical staff and one member of the nursing team responded, representing a 50 % response rate. All seven participants completed the questionnaire in full. For the second round, all first round participants were re-contacted and invited to participate. All seven participants from the first round agreed to participate, attributing to a 100 % second round response rate and 100 % questionnaire completion rate. Participant characteristics have been described in Table 1. All participants are senior palliative care team members.

**Table 1:** Characteristics of Delphi and thematic analysis participants

	Gender	Team role	Years fully qualified experience^a^	Dual specialist training^b^
*Participant 1*	Male	Medical	>15	-
*Participant 2*	Female	Medical	>5	-
*Participant 3*	Female	Medical	>5	Palliative medicine/ oncology
*Participant 4*	Male	Medical	<5	Palliative medicine/ general medicine
*Participant 5*	Female	Medical	>5	Palliative medicine/ oncology
*Participant 6*	Male	Medical	>15	-
*Participant 7*	Female	Nursing	>15	-

^a^Number of years since completing Advanced training and Chapter of Palliative Medicine (medical participants) or the number of years since completing certifications in palliative care nursing

^b^All participants are involved in hospice, acute palliative care inpatient, consult and community care

The statements assessing journey map utility are shown in Table 2. As there was a strong overall consensus following the first round of the Delphi questionnaire, these statements were not rechallenged in a second round. However free-text fields were included to allow participants to provide any further comments.

**Table 2:** Journey map assessment: consensus by statement

Statement	Round	Consensus	Percentage (%)
The journey maps were clear and easy to understand	**1**	Agree	100
	**2**	-	-
The journey maps were user-friendly	**1**	Agree	100
	**2**	-	-
There is a benefit to conveying patient information in journey maps	**1**	Agree	100
	**2**	-	-
Presenting information in the form of journey maps synthesises a greater holistic understanding in comparison to conventional methods	**1**	Agree	100
	**2**	-	-
The journey maps assisted in gauging the benefits and outcomes of palliative care intervention^a^	**1**	No consensus	
	**2**	-	-
It is possible to identify potential pivot-points of care using journey maps and the information given	**1**	Agree	100
	**2**	-	-
Patterns in the disease trajectories could be identified	**1**	Agree	100
	**2**	-	-
Journey maps would be useful in clinical practice	**1**	Agree	100
	**2**	-	-
Journey maps would be a useful way to communicate patient information with other healthcare professionals	**1**	Agree	100
	**2**	-	-

^a^Statement not considered for round 2 as feedback from first round clearly suggestive of insufficient information to determine outcomes and benefits

### Thematic analysis

Following analysis of all free-text comments, the following themes were derived regarding the applications of the journey mapping tool: (1) design and information, (2) longitudinal care and the patient trajectory and (3) opportunities for care improvement. These are discussed with supporting quotations listed in Table 3.

**Table 3:** Quotations supporting thematic analysis

Component	Quotations
*Theme 1: Design and information*	
Aspects of the interface	*“It provided a good pictorial representation, once I developed more experience and understanding of the map. It was relatively easy to understand”* – Participant 6, Journey Map Assessment *“the interface was clunky … Hovering over a button did not necessarily yield information for that button”* – Participant 5, Journey Map Assessment
Catering information needs	*“While this is a good idea, the amount of information in the map needs to be refined to be user friendly and practical.”* – Participant 5, Journey Map Assessment *“All the data was useful although inclusion of symptom management outcomes would help gauge the effectiveness of pall care input.”* – Participant 6, Journey Map Assessment
*Theme 2: Longitudinal care and the patient trajectory*	
Longitudinal care and the patient trajectory	*“The map was useful to gain insight to the longitudinal needs and barriers for which the patient experience might have been improved”* – Participant 1, Journey Map 1 *“It provided a good overview of the trajectory focus rather than being event focused as I would be in a traditional history. It provided me with a sense of the pattern of illness and insight into key points and the commentary key issues at that point.”* – Participant 1, Journey Map Assessment
*Theme 3: Opportunities for care improvement*	
Identifying barriers and missed opportunities for care	*“There were several missed triggers (e.g. disease progression into incurable stage with physical symptomatology, emotional distress) prior to eventual trigger for referral.”* – Participant 4, Journey Map 3 *“Times pain and deterioration noted in multiple onc*[ology] *clinic notes, and also the potential benefits/roles for early referral to PC, however not initiated at these times.”* – Participant 2, Journey Map 4
Clinical applications	*“Overall has great potential to be used in clinical care of patients and their families and give health providers an overview of the patient which is not possible in conventional medical histories…I can see that pivot points are evident that are pro-active and would impact clinical outcomes.”* – Participant 1, Journey Map Assessment *“It provides a good overview of service delivery and would therefore be useful for health economics/service planning”* – Participant 4, Journey Map Assessment

#### Theme 1: tool design and information

A large determinant of the practicality of the tool relates to its design. Participants provided feedback regarding the design and informational elements of the journey mapping tool used in this study.

##### Aspects of the interface

Participants found that there were certain elements of the journey maps that limited functionality of the tool, however these were associated with the specific design of the tool interface, rather than the actual components underlying the journey mapping approach. Participants responded well to the concept of a visual representation of patient information and the timeline view of care that was constructed.

##### Catering information needs

Given the palliative care specific focus on patient care presented in these maps, participants found that at times there was an excess of unnecessary information and insufficient palliative care appropriate information. The absence of objective measures of quality of life also restricted the ability of participants to determine whether outcomes were improved as a result of interventions.

#### Theme 2: Longitudinal care and the patient trajectory

The benefits of conveying patient information and healthcare encounters in the form of journey maps were also recognised. Journey maps provided a patient-centred focus of care that characterises patient healthcare interactions within a longitudinal trajectory rather than individual care episodes as is standard in conventional medical records. In doing so, patterns of the disease trajectory and also patient decline can be mapped to provide more proactive patient care.

#### Theme 3: Opportunities for care improvement

The benefits of having the journey mapping tool and its utility if incorporated into patient care were also explored, with participants noting numerous possible applications and opportunities to improve patient care.

##### Identifying barriers and missed opportunities for care

By framing the patient healthcare experience as a longitudinal and continuous journey, participants were able to recognise missed opportunities to address barriers and initiate more timely palliative care.

##### Clinical applications

Participants also noted that journey maps were a useful tool for identifying gaps in care provision and underlying barriers to initiation and delivery. This could assist clinicians with recognition of pivot-points and opportunities to enhance care by pre-emptively managing issues. Additionally, journey maps presented possible applications as a review or screening tool to evaluate patient care needs and enable better patient-centred care practices both in the clinical and research setting.

Findings from both the modified Delphi and thematic analysis appeared congruent, with the consensus consistent with emerging themes.

## Discussion

Journey mapping is a novel approach to reviewing patient healthcare interactions over time and across care settings to identify potential pivot points, which in turn can facilitate timely healthcare and promote proactive delivery of patient-centred care. Our research has focused on palliative care as the model to explore this approach, especially given its importance in an ageing population and considering many aspects of care are ubiquitous to this cohort.

Variation and inconsistencies in palliative care initiation and delivery have limited the applicability and role of research in informing evidence-based practice [6]. A journey map approach may provide one solution to address these challenges. The journey mapping tool used in this study was found to enable a patient-centred focus to the clinician’s perspective, increasing opportunities to pro-actively identify pivot-points and deliver more effective patient care.

In comparison to conventional medical records, journey maps link patient healthcare encounters longitudinally, promoting continuity and a holistic understanding of care across settings and over time. As described in conceptual studies, journey maps offer a perspective that takes into account the more dynamic and multidimensional aspects of healthcare interactions to facilitate enhanced insight into the patient experience within medical research [10, 18]. This enables a more integrated interpretation and awareness of individual episodes of care and how these contribute to a patient’s overall health and their interaction with health services. Our participants also noted that journey mapping enabled greater emphasis on particular patient outcomes that may be difficult to observe or measure using conventional research methods. The journey mapping tool was also able to highlight gaps in care and facilitate recognition of patterns of disease progression and deterioration with a greater emphasis on patient needs and experiences.

The use of the journey mapping approach has further enabled identification of barriers and potential biases to providing effective care. This study confirms that journey mapping as a tool is effective at identifying specific barriers and trends in care provision and increase opportunities for care providers to pro-actively and appropriately address these.

Journey maps have traditionally been used in research to review and analyse the consumer experience and provide feedback on avenues for development [7]. Our panel consensus affirmed that journey mapping had applications as a clinical audit tool to identify gaps in care and opportunities for improvement when used to assess retrospective patient experiences. This is consistent with known utilities of previous journey mapping tools. Other identified benefits included potential to achieve better collaboration between healthcare providers, enabling smoother transitions of care and improving communication between healthcare providers and patients.

Limitations of this study include the design and interface of the journey mapping tool. Following a thorough search, pre-existing journey mapping software and tools were considered inappropriate for this study as they were oversimplified, unable to convey complex information appropriately and not designed for use in a medical setting. Consequently, self-designing a tool was considered the most suitable approach. The technical limitations identified did not reflect the utility of the journey mapping paradigm.

The retrospective nature of this study prohibits direct patient feedback. Consequently, the patient and caregiver perspective, including quality of life and symptom burden experienced were not well represented. Future research utilising a prospective approach with patient and caregiver involvement is needed to address these research gaps.

The response rate to the initial Delphi questionnaire was only 50 % due to time constraints and limited ability to accommodate delayed responses, however the response rate to the second Delphi questionnaire was 100 %. While this does limit the diversity of responses, it suggests good retention and engagement of involved participants with meaningful contributions.

The size of the Delphi panel in this study was seven. Studies have noted that smaller panels are still able to provide effective and reliable results and a minimum panel size of seven is considered suitable in most cases [19, 20]. Our modified approach complied with this. Despite being a single institution study, the participants come from a diverse clinical background covering multiple domains of specialty palliative care, henceforth reducing potential bias. This study demonstrates that there is a role for journey mapping in clinical practice, however, considerations must be made for future design. Given the volume of patient data available, the amount of information presented needs to be appropriately moderated to provide clarity and best utilisation of the resources available. With the gradual transition of most health services from paper medical records to electronic medical records, the inclusion of a journey mapping tool into clinical practice is becoming more feasible. As medical technology continues to grow, the potential for incorporation of artificial intelligence, machine learning and big data into journey maps could be the key to providing pro-active, holistic patient-centred care that pre-emptively anticipates patient needs.

This study is one of the first to use a journey mapping tool in clinical practice to explore the healthcare journey and patient experience on a larger scale. The maps were used to depict a more fluid and continuous interpretation of the patient healthcare experience which enabled a more holistic and patient-centred analysis of palliative care provision. Furthermore, this is one of the first medical journey mapping studies to consider and propose potential pivot-points and opportunities for changes in the delivery of care. The use of journey maps can enhance the holistic patient healthcare experience and enable better patient-centred care not only in the palliative care setting, but also more broadly across healthcare from both a research and clinical practice perspective. Further application studies in other contexts are required.

## Data Availability

The datasets generated and/or analysed during the current study are not publicly available due to the confidential nature of the patient data, but are available from the corresponding author on reasonable request.
